# Search for natural products from actinomycetes of the genus *Nocardia*

**DOI:** 10.1007/s11418-024-01833-y

**Published:** 2024-08-02

**Authors:** Yasumasa Hara

**Affiliations:** Faculty of Agriculture, Kagawa University, 2393 Ikenobe, Miki, Kagawa 761-0795 Japan

**Keywords:** Natural products, *Nocardia*, Single culture, Co-culture

## Abstract

The genus *Nocardia* are gram-positive bacteria, many of which possess pathogenicity and infect human lungs, skin, brain, and other organs. Since research on the genus *Nocardia* has not progressed as rapidly as that on the genus *Streptomyces*, the genus *Nocardia* is considered a useful undeveloped resource for exploring natural products. On the other hand, when the genus *Nocardia* infects the human body, the strains are attacked by immune cells such as macrophages. Therefore, we suggested a new method for screening natural products by culturing the genus *Nocardia* in the presence of animal cells. In this review, we describe our recent results in searching for natural products from the genus *Nocardia*.

**Graphical abstract:**

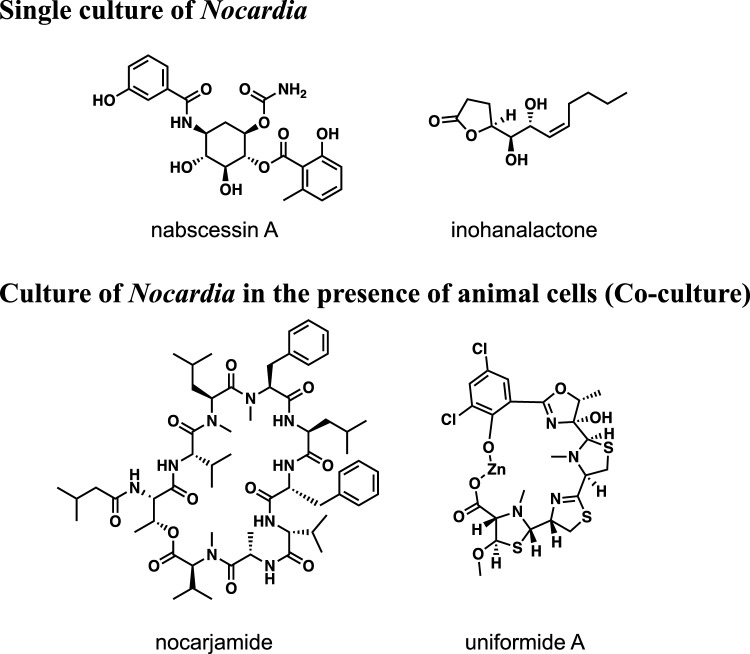

## Introduction

Actinomycetes produce various natural products with biologic activities, some of which have contributed to the development of novel medicines. In particular, many natural products have been isolated from the genus *Streptomyces*; however, there is increasing interest in the isolation of natural products from other under-exploited actinomycetes.

The genus *Nocardia* is a gram-positive bacterium, a member of actinomycetes, widely distributed in soil and water in the environment. The genus *Nocardia* consists of approximately 120 species, some of which are known to infect animals and plants and are found in the lungs, skin, brain, and other organs in humans. The study of the genus *Nocardia* began in 1888 with the isolation of the first *Nocardia* actinomycete, *Nocardia farcinica*, by Edmond Nocard [1]. Some secondary metabolites have been reported from the genus *Nocardia* [2]. Recently, terpenibactin A was isolated from *Nocardia terpenica* IFM 0406 [3], amamistatin C was isolated from *Nocardia altamirensis* DSM 44997 [4], and nocaviogua A was isolated from *Nocardia* sp. XZ19_369 [5]. Although these metabolites have been isolated, research on the genus *Nocardia* is not as advanced as that on the genus *Streptomyces*. The genome sequences of several *Nocardia* strains have been reported, but detailed analyses of their gene functions have not been performed [6].

We focused on actinomycetes of the genus *Nocardia* as a new resource in searching for natural products, in collaboration with the Medical Mycological Research Center, Chiba University. We cultured the genus *Nocardia* in various media as a single culture and in the presence of animal cells as a co-culture to search for new natural products. Herein, we describe our recent results in searching for natural products from the genus *Nocardia*.

### Single culture of *Nocardia* sp.

In this study, strains were selected among 76 strains belonging to the genus *Nocardia*, obtained from the Medical Mycology Research Center, Chiba University. A phylogenetic tree for these strains was constructed based on 16S rRNA sequence analysis using two analytical software programs, Clustal X [7] and MEGA [8]. In this tree, the strains were classified into nine clades. Biosynthetic gene clusters were examined in a gene analysis of the genus *Nocardia* using antiSMASH [9]. Based on these clades and the number of biosynthetic gene clusters, thirteen strains (*Nocardia abscessus* IFM 10029^T^, *Nocardia africana* IFM 10147^T^, *Nocardia anaemiae* IFM 0323^T^, *Nocardia arthritidis* IFM 10035^T^, *Nocardia asiatica* IFM 0245^T^, *Nocardia exalbida* IFM 0803^T^, *Nocardia inohanensis* IFM 0092^T^, *Nocardia kruczakiae* IFM 10565^T^, *Nocardia sienata* IFM 10088^T^, *Nocardia terpenica* IFM 0706^T^, *Nocardia transvalensis* IFM 0333^T^, *Nocardia vinacea* IFM 10175^T^, and *Nocardia yamanashiensis* IFM 0265^T^) were selected for small-scale culturing. After the 13 selected strains were cultured on four different liquid media (modified Czapek-Dox (mCD) [10], nutrient broth (NB) [11], Waksman [12], and Yeast–Malt–Glucose (YMG) [13]) at 28 °C with rotary shaking at 160 rpm, the culture extracts were measured by LC–MS. As a result of the LC–MS analysis, we focused on the extracts of *N. abscessus* IFM 10029^T^ and *N. inohanensis* IFM 0092^T^ cultured in mCD medium.

## Nabscessins A-C from *Nocardia abscessus* IFM 10029^T^

**Fig. 1:**
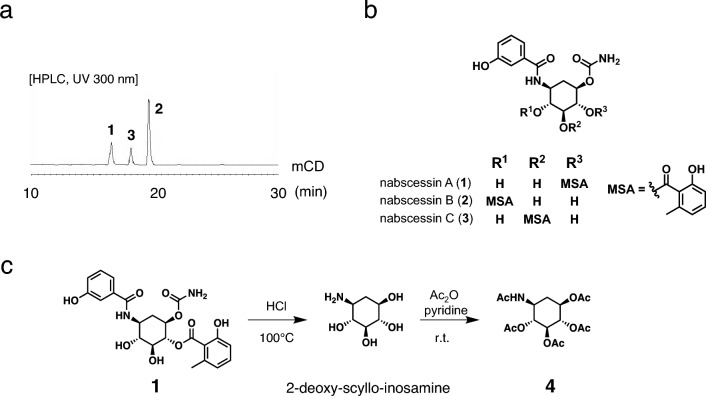
**a** *Nocardia abscessus* extracts from culture broth in modified Czapek-Dox (mCD) medium. **b** Structures of **1**–**3**. **c** The determination of absolute configuration of **1**

In the extracts of *N. abscessus* IFM 10029^T^ cultured in mCD medium, three characteristic peaks were observed. The UV absorption and the MS spectra of the three peaks were almost identical, even though the compounds represented by the three peaks exhibited distinct retention times (Fig. 1a). A large-scale culture (2.0 L) of *N. abscessus* IFM 10029^T^ was performed in mCD medium for 1 week at 28 °C with rotary shaking at 160 rpm. After centrifugation of the culture, the supernatant and a methanol extract of the mycelia were combined and subjected to partitioning between ethyl acetate (EtOAc) and water. The EtOAc fraction was subjected to fractionation by silica gel column chromatography and reverse-phase HPLC separation to obtain three new compounds, designated as nabscessins A-C (**1**–**3**) [14, 15] (Fig. 1b). Based on the NMR and MS spectra, **1**–**3** were identified as new aminocyclitol derivatives with 3-hydroxybenzoic acid, 6-methylsalicylic acid (6-MSA), and cyclohexane ring moieties. Compounds **1**–**3** were isomers with different positioning of 6-MSA. Compounds **1** and **2** were reported as new compounds in 2016 [14], but these absolute configurations had not been determined. To elucidate the absolute stereochemistry of **1**–**3**, compound **1** was converted to a 2-deoxy-*scyllo*-inosamine pentaacetyl derivative (**4**) by hydrolysis and acetylation (Fig. 1c), considering that the specific rotations of both enantiomers of **4** have been reported in the literature [16]. The spectral data of the resulting pentaacetyl compound **4** was identical to those previously reported [16], establishing the absolute configuration of **1 **[15]. Since nabscessins A-C were obtained from the same *Nocardia* strain, the absolute configurations of **1**–**3** were presumed to be the same. This inference was supported by the absolute configuration of **2** by total synthesis achieved in 2018 by Ma X. et al*.* [17].

**Fig. 2:**
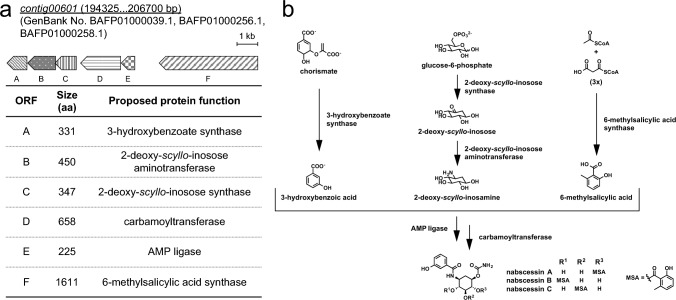
**a** The putative biosynthetic gene cluster of nabscessins A-C. **b** The proposed biosynthetic pathway for nabscessins A-C

To search for the biosynthetic gene clusters of **1**–**3**, MiGAP [18] and BLASTP [19] analyses of the draft genome of *N. abscessus* suggested the presence of 6 open reading frames, including the gene encoding 2-deoxy-*scyllo*-inosose synthase, which were possibly involved in the biosynthesis of the nabscessins (Fig. 2a) [15]. Based on the structure of this putative gene cluster, the pathway for biosynthesis of the nabscessins was proposed, as shown in Fig. 2b [15]. The expression levels of these putative biosynthesis genes for *N. abscessus* cultured in mCD medium and Waksman medium were compared by RNA-seq. Notably, **1**–**3** were produced in mCD medium but not in Waksman medium. The RNA-seq analysis revealed that the expression of the genes coding for the six biosynthetic enzymes (A-F) was 30-fold higher in mCD medium than in Waksman medium. The results showed that **1**–**3** are produced in the proposed biosynthetic pathway [15].

Compounds **1** and **2** with a aminocyclitol moiety showed antifungal activity against *Cryptococcus neoformans*, with IC_50_ values of 32 and 16 μg/mL, respectively [14].

## Inohanalactone from *Nocardia inohanensis* IFM 0092^T^

**Fig. 3:**
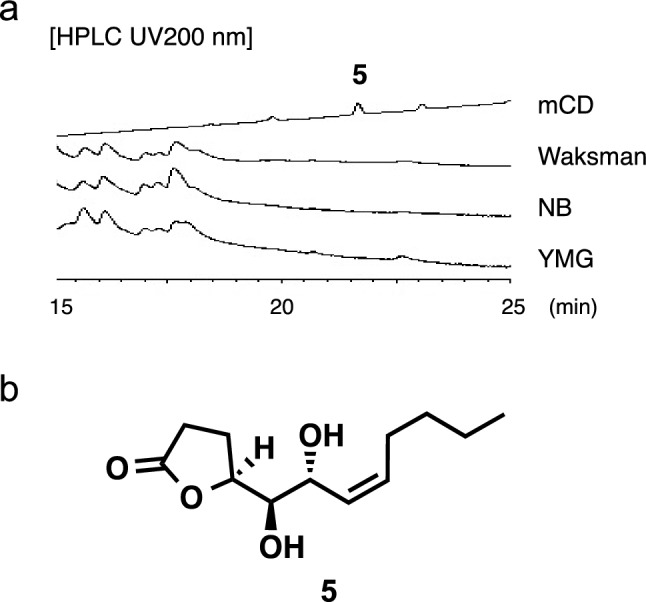
**a** Comparison of *N. inohanensis* extracts from culture broth in four media (mCD, Waksman, NB, and YMG). **b** Structure of **5**

In the extracts of *N. inohanensis* IFM 0092^T^ cultured in mCD medium, one characteristic peak was observed by HPLC. This peak was preferentially produced in the mCD medium and not in the other three media (Fig. 3a). A large-scale culture (2.0 L) of *N. inohanensis* IFM 0092^T^ was cultured in mCD medium for 2 weeks at 28 °C with rotary shaking at 160 rpm. After cultivation and partitioning with ethyl acetate, the resulting EtOAc layer was fractionated by octadecylsilyl (ODS) column chromatography to obtain the new compound, designated as inohanalactone (**5**) [20]. Based on the analysis of various spectral data, **5** was found to be a new compound with a *γ*-butyrolactone skeleton (Fig. 3b). A related compound, pseudonocardide A, with a different carbon chain length in the side chain, was isolated from the marine-derived actinomycete strain *Pseudonocardia* sp. YIM M1366912 [21]. Compound **5** likely has the same absolute configuration as pseudonocardide A (4*S*, 5*S*, 6*R*) because they exhibit similar optical rotation values. Compound **5** showed no cytotoxicity against human gastric adenocarcinoma AGS cell lines at 50 μg/mL [20].

## Co-culture of *Nocardia* sp.

Microbial co-culture is a method of culturing two or more kinds of microorganisms in the same environment, thereby potentially activating the biosynthetic genes responsible for the production of secondary metabolites [22]. This microbial co-culture is inspired by naturally occurring microbial communities, where microbial interactions through secondary metabolites are related to chemical defense and other various phenomena.

When bacteria of the genus *Nocardia* infect the human body, they are attacked by immune cells such as macrophages. Thus, *Nocardia* and macrophages can interact. We focused on this phenomenon and hoped that by mimicking and exploiting this phenomenon, the biosynthetic genes of *Nocardia* could be activated and new secondary metabolites could be obtained from *Nocardia*. Prior to our study [23], there was no report on the search for natural products using a co-culture system comprising microorganism-animal cell interactions. Therefore, we investigated the production of secondary metabolites using a co-culture in which the genus *Nocardia* is cultured in the presence of an animal cell line. The genus *Nocardia* receives supplementation with macrophages in the initial infection state [24]. It has been reported that *Nocardia asteroides* GUH-2 infected the mouse macrophage-like cell line J774.1 and induced changes in cell morphology [25] and that *N. farcinica* IFM 10152 was infected by J774.1 to show nocobactin NA-induced cell reduction [26]. Therefore, the mouse macrophage-like cell line J774.1 was selected for co-culture.

## Dehydropropylpantothenamide and nocarjamide from *Nocardia tenerifensis* IFM 10554^T^ in the presence of J774.1

The strains for the co-culture study were initially selected from 76 strains belonging to the genus *Nocardia*. A phylogenetic tree of the genus *Nocardia* was constructed using two analytical software programs (Clustal X and MEGA) and the nocobactin-related biosynthetic gene cluster [26] as an index. The strains were classified into five clades. Based on these clades and the number of biosynthetic gene clusters, six strains (*Nocardia altamirensis* IFM 10819^T^, *Nocardia mexicana* IFM 10801^T^, *Nocardia otitidiscaviarum* IFM 0239^T^, *Nocardia tenerifensis* IFM 10554^T^, *Nocardia terpenica* IFM 0706^T^, and *Nocardia vulneris* NBRC 108936^T^) were selected [23].

Culture conditions for the six selected strains were examined in the presence or absence of J774.1 using a combination of six different media, two temperatures (28 or 37 °C), two air compositions (atmosphere or 5% CO_2_), two containers, and two shaking conditions (static or rotary shaking). LC–MS analyses of the culture broth extracts obtained under various conditions revealed that multiple peaks were selectively exhibited by the extract of *N. tenerifensis* IFM 10554^T^ cultured in the presence of J774.1. The ratio of the cell numbers of *N. tenerifensis* IFM 10554^T^ and J774.1 was also examined under various conditions, demonstrating that the selective LC–MS peaks were present for the extracts obtained from the culture at a ratio of 10:1 in mCD medium.

**Fig. 4:**
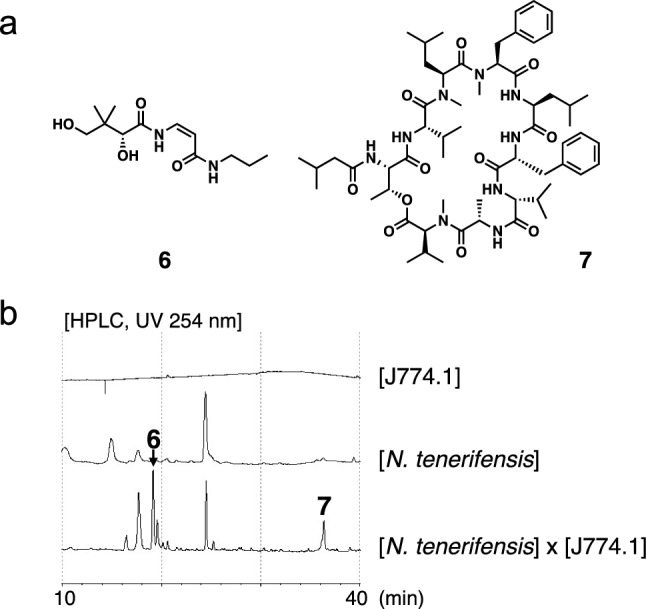
**a** Structures of **6** and **7**. **b** Comparison of the extract of *N. tenerifensis* cultured in the presence of J774.1 with the single-culture extracts of *N. tenerifensis* and J774.1 in mCD medium

A large-scale co-culture (7.3 L) of *N. tenerifensis* IFM 10554^T^ in the presence of J774.1 in mCD medium at a cell number ratio of 10:1 was performed at 28 °C in 175 cm^2^ cell culture flasks under static conditions for 2 weeks in air. After cultivation and partitioning with ethyl acetate, the resulting EtOAc layer was fractionated by ODS column chromatography and reverse-phase HPLC to yield two new compounds (**6** and **7**), named dehydropropylpantothenamide [23] and nocarjamide [27], respectively (Fig. 4a). HPLC revealed that **6** and **7** were produced under co-culture conditions in the presence of J774.1 but not under single-culture conditions in the absence of J774.1 (Fig. 4b) [23, 27].

Based on the NMR and MS spectra, **6** was identified as a new pantothenic acid derivative containing *Z*-olefin and two amides. To elucidate the absolute stereochemistry, **6** was synthesized from d-pantothenic acid calcium salt in six steps according to a previously reported method by Nicolaou K.C. et al*.* [28]. The optical rotation and CD spectrum of natural compound **6** corresponded well with those of synthetic **6** (2*R*). Therefore, the configuration at position 2 was determined to be the *R* configuration [23].

Compound **7** was revealed to have the molecular formula C_60_H_93_N_9_O_11_ by high-resolution ITTOFMS (obsd. *m/z* 1138.6897 [M + Na]^+^). Although the NMR spectral data in CDCl_3_ suggested that **7** has 18 amino acids in its substructure, the molecular weights based on these 18 amino acids significantly differed from the molecular weights estimated using the MS data. The^1^H NMR of **7** was measured in DMSO-*d*_6_, showing that the number of signals in DMSO-*d*_6_ was reduced by approximately half compared with that in CDCl_3_. These results suggested that the two conformers of **7** were observed at a ratio of almost 1:1 in CDCl_3_, whereas only one conformer of **7** was observed in DMSO-*d*_6_. The NMR analysis in DMSO-*d*_6_ suggested the presence of multiple residues, namely one alanine (Ala), one leucine (Leu), one phenylalanine (Phe), one threonine (Thr), two valines (Val^1^ and Val^2^), one *N*-methyl leucine (MeLeu), one *N*-methyl phenylalanine (MePhe), one *N*-methyl valine (MeVal), and one 3-methylbutanoic acid (MBA). The HMBC spectrum of **7** in DMSO-*d*_6_ showed that all nine amino acids were connected by eight amide bonds to yield a sequence of Thr-Val^2^-MeLeu-MePhe-Leu-Phe-Val^1^-Ala-MeVal. Additionally, HMBC correlations implied that an *N*-terminal Thr was connected to MBA by an amide bond, whereas a C-terminal MeVal was connected to the Thr by an ester bond, suggesting a planar structure for **7**. Furthermore, when MS/MS analysis was conducted using a positive ion peak *m/z* 1138 [M + Na]^+^ as the precursor ion, all product ions generated by the sequential removal of each amino acid comprising compound **7** were observed. These results were also consistent with the planar structure of **7**. The absolute configuration of the amino acids in **7** was determined using the advanced Marfey’s method [29]. Based on the results of LC–MS, **7** was revealed to consist of l-Ala, L-Leu, D-Phe, L-Thr, L-MeLeu, L-MePhe, and L-MeVal. Furthermore, the two valines (Val^1^ and Val^2^) consist of one D-Val and one L-Val, although it was unclear which one is D or L. The absolute configurations of the two valines were firmly established by an X-ray crystallographic analysis of **7**. X-ray analysis revealed that two conformers are present in a 1:1 ratio in the crystalline state of **7**. As a result, the whole structure of nocarjamide was concluded (Fig. 4a) [27].

We have previously screened natural resources against the Wnt signal, which plays a role in vital phenomena, such as tissue formation and differentiation/proliferation [30], and we have isolated various natural products to regulate the Wnt signal [31, 32]. Therefore, we evaluated **7** against the Wnt signal including β-catenin and c-myc. β-catenin is an important molecule in the Wnt signal pathway and increased β-catenin leads to up-regulation of Wnt signal activity, leading to increased c-myc protein which is a target protein of the Wnt signal pathway. As a result, the TOPFlash luciferase assay system [33] and western blot analysis showed that **7** has an activating effect on the Wnt signal by increasing protein levels of not only β-catenin but also c-myc [27].

For pathogenic microorganisms, evading macrophage-mediated cellular immunity is critical for attacking the host. For instance, the production of nocobactin NA by *N. farcinica* is cytotoxic to mouse macrophages cell, suggesting that it is involved in the pathogenicity [26]. Thus, we examined the cytotoxicity of **7** against the mouse macrophage cell line J774.1 and observed cytotoxicity with IC_50_ values of 25 μM [34].

## Peptidolipin NA derivatives from *Nocardia arthritidis* IFM 10035^T^ in the presence of J774.1

**Fig. 5:**
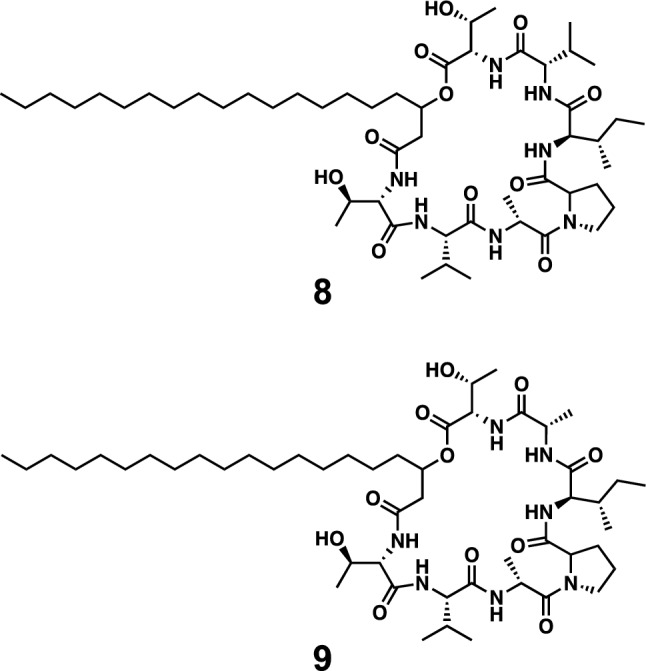
Structures of **8** and **9**

The thirteen selected strains from the phylogenetic tree of the genus *Nocardia* based on the DNA sequence for 16S rRNA [14] were cultured in mCD medium in the presence of J774.1. After centrifugation of the culture extract, the MeOH extract of the mycelium cake was partitioned between EtOAc and water. The EtOAc layer was fractionated by silica gel and ODS column chromatography and reverse-phase HPLC to yield two natural products (**8** and **9**). Based on the NMR and MS analyses, **8** and **9** were identified as two known compounds, L-Val (6) peptidolipin NA and peptidolipin NA, respectively (Fig. 5) [34]. These compounds were previously isolated from a single-culture extract of *N. asteroides* ATCC 9969 [35, 36, 46]. Compounds **8** and **9** were not isolated from the single culture but were isolated from the co-culture with J774.1 using *N. arthritidis*, which is a different species from *N. asteroides*. Compound **8** showed cytotoxicity with IC_50_ values of 116 μM, whereas compound **9** showed cytotoxicity with an IC_50_ value of > 200 μM. Next, we tested these compounds for the tumor necrosis factor-related apoptosis-inducing ligand (TRAIL) resistance-overcoming activity [37]. TRAIL binds to death receptors and selectively induces apoptosis in cancer cells. However, some cancer cells such as gastric and prostate, are resistant to TRAIL-induced apoptosis [38]. When we treated the TRAIL-resistant human gastric adenocarcinoma AGS cells with TRAIL alone, most of the cells survived. However, when the cells were treated with TRIAL along with **8**, the cell viability decreased. These results indicate that **8** may activate apoptosis in TRAIL-resistant human cancer cells [34].

## Uniformides from *Nocardia uniformis* IFM 0856^T^ in the presence of J774.1

As shown above, nocarjamide and L-Val (6) peptidolipin NA, which were isolated from *Nocardia* cultured in the presence of J774.1, were found to be cytotoxic to the mouse macrophage-like cell line J774.1 [34]. We hypothesized that one of the reasons *Nocardia* produces such natural products may be to avoid being eliminated by immune cells during infection. Therefore, we investigated natural products with cytotoxic effects on immune cells of the genus *Nocardia*.

**Fig. 6:**
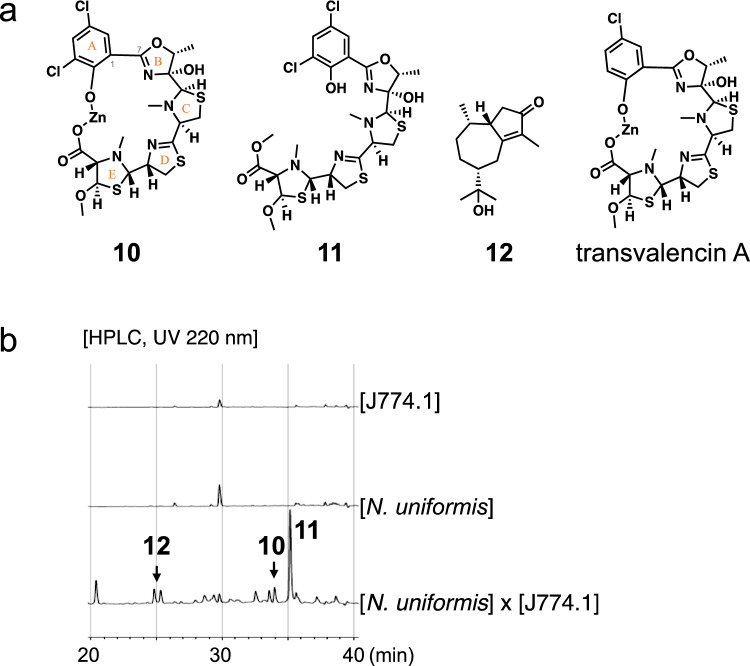
**a** Structures of **10**–**12** and transvalencin A. **b** Comparison of the extract of *N. uniformis* cultured in the presence of J774.1 with the single-culture extracts of *N. uniformis* and J774.1 in mCD2 medium

While investigating the natural products from *Nocardia* sp., we obtained the EtOAc culture extracts of 66 species in the genus *Nocardia* [39]. We focused on extracts containing cytotoxic compounds that are not produced under single-culture conditions but are produced in the presence of J774.1. From the screening for cytotoxicity against J774.1 and the LC–MS analysis of culture extracts obtained in the presence and absence of J774.1, we selected an extract of *Nocardia uniformis* IFM 0856^T^ cultured in modified Czapek-Dox 2nd (mCD2) [40, 47] medium at 28 °C in the presence of J774.1. The EtOAc extract of the large-scale culture of this strain with J774.1 was subjected to ODS chromatography and reverse-phase HPLC to obtain two new compounds (**10** and **11**) and one known compound, 3-oxoguai-4-en-11-ol (hydroxycolorenone) (**12**) (Fig. 6a) [41, 48]. Compounds **10**–**12** were not produced in the single culture of *N. uniformis* but were produced in the culture containing J774.1 in mCD2 medium (Fig. 6b) [40, 47].

Compound **10** was revealed to have the molecular formula C_23_H_26_O_6_N_4_S_3_Cl_2_Zn by HRESIMS. The NMR analysis of **10** measured in CDCl_3_ indicated the presence of a four-substituted benzene ring (A ring), a five-membered ring with two heteroatoms and an imino bond (B ring), a five-membered ring with two heteroatoms (C ring), a five-membered ring with two heteroatoms and an imino bond (D ring), and a five-membered ring with two heteroatoms, one methoxy, and one carbonyl group (E ring). The B-E rings were connected by HMBC, and the predicted partial structures of **10** corresponded well with those of transvalencin A isolated from *N. transvalensis* IFM 10065 [42, 49], suggesting that A and B rings were connected by the C1–C7 bond, and A and E rings were connected by the O–Zn–O bond. In addition, there were 34 differences between the MS spectrum of **10** and that of transvalencin A, indicating that one hydrogen atom of ring A in transvalencin A was replaced by a chlorine atom in **10**. The CD spectrum of **10** showed Cotton effects similar to that of transvalencin A, suggesting that **10** and transvalencin A have the same absolute configuration [40, 47].

Compound **11** was revealed to have the molecular formula C_24_H_30_O_6_N_4_S_3_Cl_2_ by HRESIMS. Based on 2D NMR and a comparison with the 1D NMR of **10**, the same partial structures of **10** existed in **11**, except for the O–Zn–O bond. The HMBC suggested the presence of a methyl ester moiety in ring E of **11**. The CD spectrum of **11** was similar to that of **10,** suggesting they have the same absolute configuration. Compounds **10** and **11** were named uniformides A and B [40, 47].

The structures of uniformides A and B are similar to that of transvalencin A, whose biosynthetic gene cluster was suggested by Engelbrecht A. et al. [43]. Genome analysis of *N. uniformis* JCM 3224^T^ (equivalent to NBRC 13702 and IFM 0856^T^) using antiSMASH [44] and BLAST showed that a gene cluster similar to the transvalencin A gene cluster was conserved in *N. uniformis* JCM3224^T^, and this gene cluster was suggested to be a biosynthetic gene cluster of uniformides [40, 47].

Compounds **10**–**12** were tested for cytotoxicity against J774.1 for 72 h. As a result, **10** and **11** showed cytotoxicity with IC_50_ values of 0.85 and 0.69 μM, respectively. Furthermore, experiments were conducted with various cell death inhibitors, suggesting that **11** is associated with lipid peroxidation-dependent cell death [40, 47].

Infection with pathogenic microorganisms triggers an inflammatory response mediated by innate immune cells such as macrophages. Hence, pathogenic microorganisms need to suppress the inflammatory response to attack the host. The roles of **10** and **11** in the inflammatory response were investigated using the mouse macrophage-like cell line RAW264 treated with lipopolysaccharide (LPS), and both compounds inhibited NO production. When the inflammatory response of macrophages is triggered by LPS stimulation, Toll-like receptor (TLR) 4-mediated nuclear factor-kappa B (NF-κB) signaling pathway is activated and induces the expression of inflammatory cytokines such as interleukin (IL)-6 and IL-1β [45]. In addition, when the NF-κB signaling pathway is activated, IκBα dissociates from NF-κB and is degraded by phosphorylation. The production of IκBα in the NF-κB signaling pathway involves the upstream pathways such as the PI3K/Akt signaling pathway. The effects of **11** on inflammatory responses were also investigated by RT-PCR and western blotting, showing that **11** suppressed the expression of IL-6 and IL-1β by inhibiting the degradation of IκBα in the NF-κB signaling pathway, without involving the PI3K/Akt signaling pathway [40, 47].

## Conclusion

The search for natural products from the genus *Nocardia* resulted in the isolation of new natural products, including nabscessins and inohanalactone, from single-culture extracts of *Nocardia* sp. We also suggested a new method for searching for natural products by culturing the genus *Nocardia* in the presence of animal cells and isolated new natural products such as nocarjamide and uniformides A and B.

Further natural product discovery research is in progress, aimed at discovering new natural products from the genus *Nocardia*.
